# Reducing agalsidase beta infusion time in Fabry patients: low incidence of antibody formation and infusion-associated reactions in an Italian multicenter study

**DOI:** 10.1186/s13023-024-03049-5

**Published:** 2024-02-02

**Authors:** Renzo Mignani, Claudio Americo, Filippo Aucella, Yuri Battaglia, Vittoria Cianci, Annamaria Sapuppo, Chiara Lanzillo, Fabio Pennacchiotti, Luciano Tartaglia, Giacomo Marchi, Federico Pieruzzi

**Affiliations:** 1 Nephrology, Dialysis and Transplantation, IRCCS S. Orsola Hospital, University of Bologna, Bologna, Italy; 2 Nephrology and Dialysis Unit, Pierantoni Hospital, Forlì, Italy; 3 Nephrology Unit, Ospedale Casa Sollievo della Sofferenza, San Giovanni Rotondo, Foggia, Italy; 4 Nephrology and Dialysis Unit, St. Anna University Hospital, Ferrara, Italy; 5 Regional Epilepsy Centre, Great Metropolitan Hospital, Reggio Calabria, Italy; 6 Pediatric Clinic, Department of Clinical and Experimental Medicine, University of Catania, Catania, Italy; 7 Division of Cardiology, Policlinico Casilino, Rome, Italy; 8 Nephrology Unit, Manduria Hospital, Taranto, Italy; 9 Nephrology Dialysis and Transplantation Unit, Department of Medical and Surgical Sciences, University of Foggia, Foggia, Italy; 10 MetabERN Referral Center for Lysosomal Storage Disorders, Internal Medicine Unit, University of Verona, Verona, Italy; 11 Clinical Nephrology, School of Medicine and Surgery, University of Milano, Bicocca, Italy; 12 Nephrology and Dialysis Unit, Fondazione IRCCS San Gerardo dei Tintori, Monza, Italy

**Keywords:** Anti-drug antibodies, Fabry disease, Enzyme replacement therapy, Agalsidase beta, Infusion time, Infusion-associated reactions

## Abstract

**Background:**

Fabry disease is a rare progressive X-linked lysosomal storage disease caused by mutations in the GLA gene that encodes α-galactosidase A. Agalsidase beta is a recombinant enzyme replacement therapy authorized in Europe at a standard dose of 1.0 mg/kg intravenously every other week at an initial infusion rate of ≤ 0.25 mg/min until patient tolerance is established, after which the infusion rate may be increased gradually. However, specific practical guidance regarding the progressive reduction in infusion time is lacking. This study investigated a new and specific protocol for reducing agalsidase beta infusion time in which a stable dosage of 15 mg/h is infused for the first four months, and the infusion rate is increased progressively from 15 to 35 mg/h for the subsequent four infusions. The shortest infusion time is reached after six months and maintained thereafter. The incidence of infusion-associated reactions (IARs) and the development of anti-drug antibodies were analyzed, and the disease burden and the clinical evolution of the disease at 12 months were evaluated.

**Results:**

Twenty-five of the 31 patients were naïve to enzyme or chaperone treatment at baseline and six patients had been switched from agalsidase alfa. The reduced infusion time protocol was well tolerated. Only one patient exhibited an IAR, with mild symptoms that resolved with low-dose steroids. Six patients globally seroconverted during treatment (4 with a classic phenotype and 2 with late-onset disease). All but three patients were seronegative at month 12. All patients were stable at the study’s end (FAbry STabilization indEX value < 20%); reducing infusion time did not negatively impact clinical outcomes in any patient. The perceived medical assessment showed that the quality of life of all patients improved.

**Conclusions:**

The study demonstrates that reducing agalsidase beta infusion time is possible and safe from both an immunogenic and clinical point of view. The use of a low infusion rate in the first months when the probability of onset of the development of antibodies is higher contributed to very limited seroconversion to antibody-positive status.

**Supplementary Information:**

The online version contains supplementary material available at 10.1186/s13023-024-03049-5.

## Introduction

Fabry disease is a progressive X-linked inherited lysosomal storage disorder caused by mutations in the GLA gene leading to deficient α-galactosidase (α-Gal) A activity, glycosphingolipid accumulation, and potentially life-threatening complications [1]. Since 2001 enzyme replacement therapy (ERT) has been available to treat Fabry disease. In Europe, two different compounds of ERT are available; agalsidase alfa and agalsidase beta [2–4]. The first is a recombinant enzyme of human origin authorized at a standard dose of 0.2 mg/kg body weight intravenously every other week [5]. In contrast, agalsidase beta is a recombinant enzyme derived from hamster ovary cells and is licensed for a standard dose of 1.0 mg/kg intravenously every other week [2–5]. Since 2017, an oral chaperone therapy with migalastat has been available for patients with amenable mutations able to maintain residual enzyme activity [6].

In addition to different authorized doses for the two agalsidase formulations, there are differences in terms of the duration of the infusions and the incidence of the formation of anti-drug antibodies (ADA). For agalsidase alfa, the infusion time indicated in the European Summary of Product Characteristics (SmPC) is 40 min [5]. In contrast, the SmPC for agalsidase beta has stated, since September 2022, that the initial infusion rate should be no more than 0.25 mg/min (15 mg/h) and that after the patient tolerance is established, the infusion rate may be increased gradually with subsequent infusions without specific practical guidance regarding the progressive reduction in infusion time [7]. Regarding the ADA generation issue, some reports have documented a higher development rate in male patients with nonsense mutations and treated with agalsidase beta rather than agalsidase alfa [4, 8]. The subclass of neutralizing antibodies in ADA-positive patients can reduce the efficacy of ERT in Fabry disease patients, as documented by the increase of plasma globotriaosylsphingosine (Lyso-Gb3) level or by the deterioration of renal and cardiac involvement [9–12]. Moreover, some authors have postulated that the probability of developing ADA is higher when the infusion rate is elevated [8, 13]. It has also been reported that the period of primary incidence of ADA development occurs in the first six months from the beginning of ERT for both agalsidase formulations [8, 13, 14]. Men who developed anti-αGAL immunoglobulin G (IgG) antibodies were more liable to experience infusion-associated reactions (IARs) than those who remained seronegative [8].

In order to address the knowledge gaps regarding the effect of different infusion times for agalsidase formulations, a multicenter study was started in Italy in January 2021 to test a new and specific protocol for reducing agalsidase beta infusion time till two hours of infusion time equal to 35 mg/h [15]. To note, in July 2022, we anticipated the course of this Italian multicenter study with the indication of a protocol for the reduction of agalsidase beta infusion time that could be safe, well-tolerated and with a low incidence of ADA generation [15]. In parallel, in October 2022, the European Summary of Product Characteristics for agalsidase beta was updated to state that the infusion rate may be modified in increments of 0.05 to 0.083 mg/min (increments of 3 to 5 mg/hr) with each subsequent infusion reaching a minimum infusion duration of 2 h. A further decrease of the infusion time to 1.5 h was allowed for patients without new IARs during the last 10 infusions or reported serious adverse events (SAEs) within the last 5 infusions [7].

Here, we present the results of the Italian multicenter study in which we analyzed the tolerance, the incidence of ADA seroconversion, and the clinical outcome after 12 months of algasidase beta administration according to the proposed time reducing infusion protocol.

## Patients and methods

The study’s primary endpoints were tolerance, evaluated by the incidence of IARs, and the frequency of seroconversion, evaluated by the development of ADA. A secondary endpoint evaluated disease burden and the clinical evolution of the disease during the infusion rate variations using the FAbry STabilization indEX (FASTEX) at 12 months as a measure of clinical stability [16]. Anti-drug antibodies were evaluated in all patients at baseline and monthly for 12 months thereafter. A patient-reported outcomes questionnaire was completed at the end of the study to assess the impact of reducing infusion time on quality of life.

Patients were enrolled from 11 reference centers for Fabry disease in Italy. All patients were diagnosed with Fabry disease, including genetic, α-Gal enzymatic activity and lyso-Gb3 [17]. Enrolled patients were treatment-naïve or already treated with agalsidase alfa at baseline and switched to agalsidase beta for clinical reasons. In the latter case, ADA was absent at baseline.

The study was conducted in accordance with the tenets of the Declaration of Helsinki and the requirements of the local ethics committees, and written informed consent was obtained from all participants included in the study.

### Agalsidase beta infusion protocol

The infusion protocol is detailed in Table 1. In the first phase of the protocol (stable phase), a stable dosage of 15 mg/h is infused for the first four months (eight infusions). No drugs for premedication treatment were prescribed at the beginning of treatment, partly to avoid potential corticosteroid-related adverse reactions. In the second protocol phase (escalation phase) for the subsequent four infusions, the infusion rate is increased progressively from 15 to 35 mg/h. In this way, after six months from the beginning of the treatment, the patient reaches the shortest agalsidase beta infusion time (2 h), which is maintained until the end of the study at 12th month. Anti-drug antibodies were measured in all patients at baseline, at months 3, 6, and after 1 year from the start of agalsidase beta treatment. The clinical outcome was evaluated at the end of the observational period of 12 months using the FASTEX score that evaluates the condition of clinical stability after one year of treatment.

**Table 1:** Infusion time reduction protocol

Dilution	Dose, mg/h	Duration, min	Rate, mL/h
1st phase (from 1st to 8th infusion)			
500 mL SS with 2 vials (70 mg)	15	280 (4h, 40′)	107
250 mL SS with 1 vial (35 mg)	15	140 (2h, 20′)	107
2nd phase (from 9th infusion onward)			
9th Infusion			
500 mL SS with 2 vials (70 mg)	20	210 (3h, 30′)	143
250 mL SS with 1 vial (35 mg)	20	105 (1h, 45′)	143
10th Infusion			
500 mL SS with 2 vials (70 mg)	25	168 (2h, 48′)	178
250 mL SS with 1 vial (35 mg)	25	84 (1h, 24′)	178
11th Infusion			
500 mL SS with 2 vials (70 mg)	30	140 (2h, 20′)	215
250 mL SS with 1 vial (35 mg)	30	70 (1h, 10′)	215
12th and subsequent Infusions			
500 mL SS with 2 vials (70 mg)	35	120 (2h)	250
250 mL SS with 1 vial (35 mg)	35	60 (1h)	250

*SS* saline solution

All patients were treated in a hospital setting and could move to home therapy only if eligibility criteria for home infusion were satisfied after the first 12 months of the infusion protocol.

### Infusion-associated reactions

Infusion-associated reactions (IARs) are defined as any related adverse event occurring on the infusion day during or after an agalsidase beta infusion. If an IAR develops during the escalation phase, the infusion is to be stopped and/or the infusion rate decreased, and premedication (non-steroidal anti-inflammatory medicinal products, antihistamines and/or corticosteroids) is administered. During the following infusions, the patient is to continue to receive the premedication while the maximum tolerated infusion rate is kept constant.

### Anti-agalsidase beta antibodies

Serum specimens for anti-agalsidase beta antibodies were collected at baseline (before starting agalsidase beta treatment) and at months 1, 3, 6, and 1 year from the beginning of the study. Possible and additional serum specimens were considered if collected in other stages.

Each sample was collected using a serum separator or a red top (5mL). Samples were then stored at < 20° C until the pickup. Analyses were conducted at LabCorp’s (Health Care Diagnostics Services Provider) specialized laboratories, the Monogram Biosciences Inc. facility in San Francisco, California, USA and/or at Esoterix Inc. facility in Calabasas, California, USA using enzyme-linked immunosorbent (ELISA) and confirmatory radioimmunoprecipitation (RIP) assays if positive.

To evaluate IgG, patient serum was screened for anti-agalsidase beta IgG antibody, with positive samples confirmed and quantitated within a reportable range of 100 to 204,800 as reported by LabCorp; serial two-fold dilutions are tested, starting with a 1/100 dilution of serum required to perform the assay. Antibody Titer is reported as the reciprocal of the highest serum dilution in which the antibody is considered positive by a validated cut-point. For example, a titer of 800 represents 1/8 dilution of the 1/100 achieved after 3 cycles of serial dilution (1/2 to 1/4 to 1/8), and 100 is the lowest possible titer. If anti-agalsidase beta antibodies are not detected, Antibody Status is reported as negative and antibody titer is not applicable (N/A).

Since neutralizing antibody testing is intended to further characterize drug-specific IgG, patient serum is first analyzed for agalsidase beta IgG. When agalsidase beta IgG is negative, the agalsidase beta neutralizing antibody assay is not performed.

### FASTEX and quality of life

The disease burden and the clinical evolution during the variations in infusion rate were evaluated with FASTEX calculated one year after the baseline.

Laboratory and instrumental investigations required for periodic clinical assessment were performed for all patients at baseline and at the 12th month to calculate the FASTEX at the end of the study. All patients should have performed the clinical and instrumental investigation necessary for measuring the FASTEX. The index was obtained by registration on the site https://www.fastex-online.com. As stated in the validation studies, patients were considered clinically stable if the calculated FASTEX was below 20%, representing the limit of clinical stability from a mathematical point of view [15].

In addition, a perceived medical assessment to evaluate whether the infusion time reduction had improved the patient’s quality of life, using the scale from 1 to 4 to weight its relevance with respect to quality of life [18, 19], was administered to the referral physicians (See Additional file 1: Appendix). Items on the questionnaire regarded the infusion duration, the comfort during the infusion, concern that the increase of the infusion rate could cause IARs, the dilution volume of the infusion, and the lack of premedication drugs.

## Results

The baseline characteristics of patients are shown in Table 2. Thirty-one patients (15 males and 16 females) with a confirmed diagnosis of Fabry disease were enrolled. The mean age was 52.4 years. Twenty-three patients presented a classic phenotype (11 males and 12 females), while eight (4 males and 4 females) presented a late-onset phenotype. Twenty-five patients were naïve to enzyme or chaperone treatment at baseline, while six patients had been switched from agalsidase alfa. The baseline mean plasma Lyso-Gb3 value was significantly higher in the classic group of patients than in the late-onset patients (33.7 ± 31 ng/L vs. 3.6 ± 4 ng/L, *p* < 0.01).

**Table 2:** Baseline characteristic of 31 patients

Parameter	
Age, years, mean ± SD	52.4 ± 15.1
Sex, n (%)	
Female	16 (51.6)
Male	15 (48.4)
Classic Fabry disease, n (%)	23 (74.2)
Plasma Lyso-Gb3, mean ± SD (range), nMol/L (normal value < 1.9)	33.7 ± 31.0 (3.0–115.4)
Serum α-Gal A activity, mean ± SD (range), nMol/h/mL (reference range > 3.8)	0.13 ± 1.1 (0.0–0.61)
Late-onset disease, n (%)	8 (25.8)
Plasma Lyso-Gb3, mean ± SD (range), nMol/L (normal value < 1.9)	3.6 ± 4.0 (1.5–22.3)
Serum α-Gal A activity, mean ± SD (range) nMol/h/mL (reference range > 3.8)	3.8 ± 6.7 (0.10–16.3)
Prior therapy, n (%)	
Naïve	25 (80.6)
Agalsidase alfa	6 (19.4)

### Tolerance

Most of the 31 patients enrolled tolerated the reduced infusion time protocol well. After beginning the agalsidase beta infusion protocol, an IAR was exhibited by only one patient, after the second infusion, with mild fever and pruritus that responded to the administration of oral low-dose steroids. No further IARs episodes were recorded in that specific patient or all other patients, either in the stable or in the escalation phases of protocol until the end of the study. Other than the single IAR, no AEs or SAEs were reported.

### Anti-agalsidase beta antibodies

All patients were negative for IgG ADA at baseline, including those who switched from agalsidase alfa (Table 3). During the observational study, six patients (5 males and 1 female) were globally seroconverted: four patients with a classic phenotype, while two presented a late-onset phenotype. In particular, at the 1st month, three patients were positive (with an IgG titer/dilution between 800 and 6400). At the 6th month, five patients (4 males and 1 female) were positive (with an IgG titer between 200 and 3200). At the 12th month, of the six seroconverted patients, only three males maintained seroconversions, while the remainder were negative. The neutralizing AB status of the patients with IgG positive titers ranged from 1.1 to 4.7 μL/mL.

**Table 3:** Anti-drug antibodies and infusion-associated reactions during treatment with agalsidase beta

#	Sex	Prior therapy	Genotype	Phenotype	Weight(Kg)	Anti-drug antibodies (titer)^a^							
						Baseline	1st mo	2nd mo	3rd mo	4th mo	5th mo	6st mo	12th mo
1	M	naïve	p.I354K	Classic	69	0							
2	M	naïve	p.P265L	Classic	74	0							
3	F	alfa	p.A156V	Classic	48	0							
4	M	naïve	c.1213 delA	Classic	60	0					POS3200		POS6400
5	F	naïve	c.1213 delA	Classic	56	0		IARs					
6	M	naïve	c.1213 delA	Classic	45	0			POS3200	POS800			POS800
7	F	naïve	c.1213 delA	Classic	58	0							
8	F	naïve	c.1213 delA	Classic	67	0							
9	F	naïve	p.L414Cfs*3	Classic	58	0							
10	M	alfa	p.N215S	Late-onset	83	0	POS3200	POS1600	NEG	NEG			NEG
11	F	naïve	p.S235C	Classic	64	0							
12	F	naïve	c.583insG	Classic	55	0							
13	M	naïve	c.388 A > G	Classic	70	0							
14	M	naïve	c.1284_1287delA	Classic	73	0							
15	F	alfa	p.L275H	Classic	48	0							
16	F	naïve	p.F113L	Late-onset	67	0							
17	F	naïve	p.F113L	Late-onset	66	0							
18	M	naïve	p.F113L	Late-onset	65	0							
19	F	naïve	p.F113L	Late-onset	52	0							
20	F	naïve	p.F113L	Late-onset	64	0							
21	M	alfa	p.F273S	Classic	65	0							
21	F	alfa	p.R301P	Classic	51	0							
23	F	alfa	p.G260E	Classic	79	0						POS200	NEG
24	M	naïve	c.1234 A > C	Classic	57	0							
25	M	naïve	IVS5 + 1G > T	Classic	59	0			POS6400	POS3200	POS1600	POS1600	NEG
26	M	naïve	c.1234 A > C	Classic	60	0							
27	M	naïve	c.1234 A > C	Classic	60	0							
28	F	naïve	c.1234 A > C	Classic	59	0							
29	M	naïve	p.I303F	Late-onset	83	0						POS800	POS800
30	F	naïve	p.G85D	Classic	55	0							
31	M	naïve	p.N215S	Late-onset	92	0							

^a^ The reportable range of antibody titer was 100 to 204,800

*alfa* agalsidase alfa, *F* female, *IARs* infusion-associated reactions, *M* male, *mo* month, *NEG* negative, *POS* positive Titer

### Clinical impact of reduction of infusion time

To analyze the potential effect of reducing the infusion time on clinical outcomes in the enrolled patients, we analyzed the FASTEX score at the end of the study (at the 12th month). None of the 31 patients enrolled demonstrated signs or symptoms of deterioration. Therefore, all patients were stable at the study’s end with a FASTEX value < 20%.

### Impact on quality of life

**Fig. 1:**
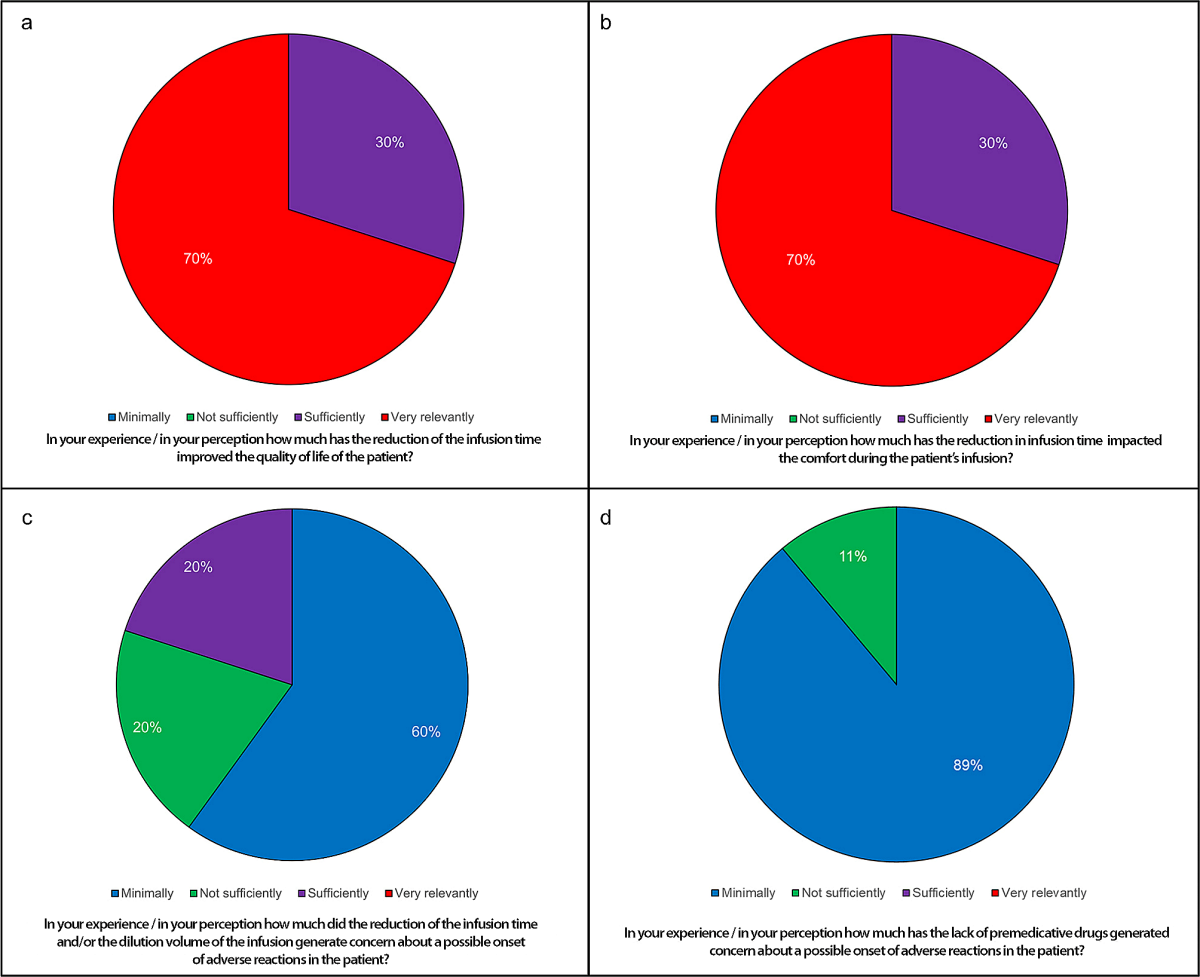
Impact of reduction in infusion time and/or dilution volume on (**a**) quality of life; (**b**) comfort during infusion; (**c**) concern about onset of adverse events, and (**d**) effect of lack of premedication drugs in the protocol on concern about the onset of adverse reactions

The perceived medical assessment of the referral physicians considered that all patients’ quality of life improved. 70% of the physicians reported that the reduction in infusion time improved the patients’ quality of life very relevantly and 30% sufficiently (Fig. 1a), with the same proportion reporting an improvement in comfort during the infusions (Fig. 1b). In addition, the reduction in infusion time and the lack of premedication were not reported as a very relevant concern regarding the possible onset of adverse reactions in patients (Fig. 1c and d).

## Discussion

The treatment for Fabry disease with ERT, with worldwide availability of more than 20 years, has undoubtedly modified the disease’s natural history, improving morbidity and increasing patient survival and quality of life. However, several aspects of ERT are still not clarified. Some aspects concern the treatment’s practical aspects, such as the infusion modality. While for agalsidase alfa, the treatment schedule is straightforward and short, for agalsidase beta, the long infusion time recommended in the SmPC negatively influences patient activities and quality of life. Besides, a faster infusion rate for agalsidase beta appears more likely to increase the chance of ADA development, possibly reducing its efficacy [2]. Moreover, we still do not know how the enzyme uptake could be with a faster infusion rate and, consequently, the possible lack of efficacy in restoring substrate degradation.

At first, an escalation dose protocol for reinstitution of agalsidase-beta therapy in Fabry disease patients with previous IgE-antibody or skin-test reactivity to the recombinant enzyme led to a successful outcome. However, in this early study only a clinical evaluation was made since ADA determination was not assessed [20].

A worldwide trend has developed to reduce infusion time indiscriminately and without clear, standardized rules. Some recent reports suggest reducing the infusion time of agalsidase beta, proposing protocols with a very low infusion time, even in the range of 40–45 min. Sanchez and co-workers have recently suggested a protocol they tested on six classic patients (5 females), where the main endpoint was clinical tolerance to a rapid and progressive reduction of agalsidase beta infusion with a target of 45 min reached after a mean of 30 months. They reported only one IAR episode in their cohort [21]. However, this work leaves many issues unanswered, such as when to start reducing infusion times, how to monitor the efficacy of agalsidase beta, and the impact of such short infusion time on the development of ADA. Along the same lines is a small experience in five classic males members of the same family, where the infusion time was progressively reduced to about 50–60 min after about 30 infusions from the beginning of treatment, reaching infusion rates of 1.57 mg/min (94 mg/h), more than 4 times the infusion rate recommended [22]. However, in this experience, neither the clinical outcome nor the potential ADA development was investigated. Of interest, Riccio et al. recently published their experience with a stepwise infusion rate escalation protocol in 53 Fabry patients mainly infused at home after the first four infusions in the hospital, in which they retrospectively explored infusion rate tolerability. However, the immune response was not evaluated in all patients (antibody measurement was implemented only in 18 patients). The authors concluded that their infusion rate escalation protocol was safe, well tolerated, and could improve patient compliance, treatment satisfaction, and quality of life [23]. Finally, in a recent Japanese post-marketing study, the rate of IARs, AEs, and SAEs was analyzed in a cohort of patients who received reduced-duration agalsidase beta infusion times of less than 90 min. There was little impact on infusion duration on safety outcomes in these patients, and no changes in antibody status were observed after infusion durations were reduced [24].

In our study, the effects of a new infusion protocol of agalsidase beta were evaluated, considering not only the safety but also the immunogenic and clinical consequences of a reduction in infusion time. With this aim, we split the protocol into two phases. In the first phase (stable phase), the infusion rate of each infusion was 15 mg/h for the initial 8 infusions, as the literature suggests that the early months of agalsidase beta infusion are the most critical for the development of ADA [8, 13, 14]. Afterward, in the subsequent 8 treatments, the infusion rate was progressively increased (escalation phase), setting a limit of infusion rate not higher than 0.8 mg/min (35 mg/h), equivalent to an infusion time of two hours for a standard patient of 70 kg. Moreover, we enrolled both treatment-naïve patients and patients previously treated with agalsidase alfa (seronegative at baseline) and patients with classic or late-onset phenotypes of both genders. In our experience, we observed only one episode of mild IAR and a cumulative incidence of seroconversion in 6 patients (19.4%). Four of those patients presented a classic and two a late-onset phenotype. Three patients became negative at the 12th month time point. The incidence of ADA in our study can be compared with data from the literature. The incidence of seroconversion in patients treated with agalsidase beta with standard infusion time, according to the SmPC, ranges between 68% and 90% [13, 25–27]. In our study, only six patients developed ADA; 5 were males (33%) and 1 female. In 50% of them, the antibodies disappeared after 1 year from starting agalsidase beta.

We did not observe the correlation reported in the literature between the absence of enzyme activity and seroconversion. Moreover, both the residual α-Gal A activity and the type of mutation were not enough to predict antibody development in an individual patient [28]. Besides, as already reported in the literature [11], treatment-naïve patients had a lower antibodies comparison (17%) than patients previously treated with agalsidase alfa (25%). Only one of the patients seroconverted was a female.

From a clinical point of view, all patients were stable according to FASTEX evaluation at the end of the infusion protocol due to an improvement or stabilization of the clinical parameters. This result is probably due to the fact that almost all patients were starting ERT for the first time, and that the infusion time reduction protocol resulted in a significant treatment benefit.

Finally, the referral physicians perceived assessment of the patients quality of life confirmed the beneficial effect of the reduced infusion duration in terms of improved overall quality of life and compliance with the infusions.

The main limitation of this study is the absence of a control group of patients treated with the conventional infusion schedule rate, as illustrated in the SmPC. Therefore, to counter this, the results of ADA development in the enrolled patients were compared with literature data. We also acknowledge the relatively small number of patients in the study, and the limited follow up duration. In addition, the lack of Lyso-Gb3 determination in all patients during the infusion protocol does not allow an analysis of Lyso-Gb3 values in seroconverted patients. However, despite these limitations, we believe that our results provide a useful basis for further investigation.

## Conclusions

Our study demonstrates that reducing agalsidase beta infusion time is possible and safe, either from an immunogenic or clinical point of view. The patient seroconversion is very limited by maintaining a low infusion rate in the first months, when the probability of onset of the development of antibodies is higher. The reduction of agalsidase beta infusion time, compared to the international SmPC schedule, does not seem to increase the patient immunogenicity, as shown by the low incidence of ADA generation despite a higher infusion rate. Furthermore, we believe that a shorter infusion time for agalsidase beta can be applied in a larger cohort of patients, but only if the immunogenicity response to a higher infusion rate is carefully monitored. Future studies are warranted to analyze the long-time safety and the effects of a shorter infusion time on the overall clinical outcome in Fabry disease patients.

### Electronic supplementary material

Below is the link to the electronic supplementary material.

**Additional file 1:** Appendix: quality of life survey

## Data Availability

The datasets used and/or analyzed during the current study are not openly available due to reasons of sensitivity and are available from the corresponding author upon reasonable request and with the permission of the patients.

## Abbreviations

ADA: Anti-drug antibodies
AE: Adverse event
α-Gal: α-galactosidase A
ERT: Enzyme replacement therapy
Lyso-Gb3: Globotriaosylsphingosine
IARs: Infusion-associated reactions
IgG: Immunoglobulin G
SAE: Serious adverse event
