# Supplementary material for: Reducing agalsidase beta infusion time in Fabry patients: low incidence of antibody formation and infusion-associated reactions in an Italian multicenter study
Source: Orphanet J Rare Dis. 2024 Feb 2;19:38. doi: 10.1186/s13023-024-03049-5 (PMC10835838; doi:10.1186/s13023-024-03049-5)
Supplement: Supplementary file 1 — Additional file 1: Appendix: quality of life survey [file 13023_2024_3049_MOESM1_ESM.pdf]

SURVEY QoL

Perceived medical assessment of the impact on the patient's QoL using the scale from 1 to 4 to weight its relevance with respect to QoL (Polit & Beck, 2006; Lawshe, 1975)

Method of administration: Telephone interviews

EVALUATION CRITERIA

FOR EACH ITEM, AN EVALUATION OF THE IMPACT ON THE QUALITY OF LIFE FROM 1 TO 4 (LIKERT SCALE) IS REQUESTED, WHERE:

- 1 MINIMALLY
- 2 NOT SUFFICIENTLY
- 3 SUFFICIENTLY
- 4 VERY RELEVANTLY

1. IN YOUR EXPERIENCE / IN YOUR PERCEPTION HOW MUCH HAS THE REDUCTION OF THE INFUSION TIME IMPROVED THE QUALITY OF LIFE OF THE PATIENT?

2. IN YOUR EXPERIENCE / IN YOUR PERCEPTION HOW MUCH HAS THE REDUCTION IN INFUSION TIME IMPACTED THE COMFORT DURING THE PATIENT'S INFUSION?

3. IN YOUR EXPERIENCE / IN YOUR PERCEPTION HOW MUCH DID THE REDUCTION OF THE INFUSION TIME AND/OR THE DILUTION VOLUME OF THE INFUSION GENERATE CONCERN ABOUT A POSSIBLE ONSET OF ADVERSE REACTIONS IN THE PATIENT?

4. IN YOUR EXPERIENCE / IN YOUR PERCEPTION HOW MUCH HAS THE LACK OF PREMEDICATIVE DRUGS GENERATED CONCERN ABOUT A POSSIBLE ONSET OF ADVERSE REACTIONS IN THE PATIENT?

Lawshe, C. (1975). "A quantitative approach to content validity." Pers Psychol 28: 563-575.

Polit, D. F. and C. T. Beck (2006). "The content validity index: are you sure you know what's being reported? Critique and recommendations." Res Nurs Health 29(5): 489-497.
